# Identification tags and learners’ situational awareness during high-fidelity simulation

**DOI:** 10.5116/ijme.56ed.1060

**Published:** 2016-03-26

**Authors:** Issam Tanoubi, Marie-Ève Bélanger, L. Mihai Georgescu, Roger Perron, Jean-Francois Germain, Arnaud Robitaille, Pierre Drolet

**Affiliations:** 1 Department of Anesthesiology, Centre d Apprentissage des Attitudes et Habiletes Cliniques, Universite de Montreal, Montreal, Quebec, Canada; 2 Centre d Apprentissage des Attitudes et Habiletes Cliniques, Faculty of Medicine, Universite de Montreal, Montreal, Quebec, Canada

**Keywords:** Identification tags, situational awareness, high-fidelity simulation

## Introduction

Simulation-based medical education is an integral part of the curriculum of many specialties. Simulation allows participants to develop and practice technical skills useful to the management of rare and urgent clinical situations in a safe and supportive learning environment.^1^ Several points are deemed essential to the promotion of optimal learning during simulation. One, which was emphasized by Issenberg and associates, stated that acquiring experience in medicine was governed by the learners’ commitment to a simulation’s realistic environment.^2^ Obtaining and maintaining the commitment of participants in a high-fidelity simulation scenario is then essential for a better learning experience. One way to foster a learner’s commitment toward a simulated environment is to help the learner gain a greater awareness of the environment’s characteristics. With better situational awareness, participants can more easily understand the various elements and the complexity of the environment, and then anticipate the human and material resources that might be needed. This could allow students to engage in better clinical decision making.

Situational awareness has been measured by quantitative scales in studies conducted in the aviation and maritime fields, but additional adjustment and validation of these scales are still required.^3^^,^^4^ Moreover, specific factors affecting the situational awareness remain unstudied.

Here, we undertook this research to assess whether wearing badges or name tags stating each participant’s function and posting a sign to clearly inform students of the scenario’s location during the high-fidelity simulation could lead to greater awareness of each learner’s role and contribute to their commitment. We hypothesized that using badges and formally identifying the location of the scenario would enable participants to feel more committed and to better identify everyone’s role and where the action was taking place.

### Evaluating the importance of the name tags and place identification

We used a pretest-posttest design to achieve our study goals. Our study took place within a planned simulation training course for 25 anesthesiology residents on the management of critical anesthesia situations from April 30, 2014, to June 11, 2014. Study coordinators assigned 4 to 5 participants to each of the 6 overall simulation sessions scheduled. Each session was 4 hours long and included an introduction, followed by two high-fidelity simulated scenarios with debriefing. The high-fidelity simulation was operationalized through high equipment fidelity (functionality and responsiveness of patients, manikins, and medical instruments), high environment fidelity (real world overload demands), and high psychologic fidelity (maintaining the natural ‘‘flow’’ of a clinical scenario and participant’s immersion within the scenario).^5^

One scenario was about a patient who had a massive amniotic fluid embolism that occurred after a complicated delivery and leading to a maternal cardiac arrest, and the alternative scenario was of a patient with postoperative malignant hyperthermia. The order of the two scenarios was randomized for each session. During the first scenario of the session, participants did not wear badges, and no sign that formally identified the mock location was allowed. In the second scenario, name badges and identifying signs were used systematically.

At the end of each scenario, all residents completed, using an audience response system (Turning Point, Ontario, Canada, Turning Technologies), a 7-question survey  (7-point Likert scale) to evaluate their situational awareness and their level of commitment. An evaluator, blinded to the randomization sequence, listened to the soundtrack only of the audio-video recording of the simulated scenarios in search of indicators suggesting a lack of situational awareness. We noted no differences between the groups regarding their consciousness of the location, or of the various roles played by the participants. We found that the resident’s engagement was also similar in both groups. The number of indicators of poor situational awareness, noted by the blinded evaluator during audio review of the scenario’s video recording was not significantly different between groups.

### Situational awareness during high-fidelity simulation

Situational awareness is described as the perception and understanding of complex environments by an individual, which leads to decision making. Several studies have highlighted the importance of maintaining situational awareness among participants during simulation-based education, whether it applies to health care or other fields.^6^ An enhanced situational awareness helps those involved to move toward decision making, leads to superior performance in simulation, and could potentially reduce the risk of errors.^7^

Simulations that have people playing roles other than their own are done in many different ways and with different types of participants. Some simulations in which multiple disciplines are present are done in a manner similar to our study, where participants (from only one discipline) play all of the roles, with these roles mostly being those at which they themselves are not experts. Some simulations are done as true combined team sessions, with actual clinicians in those roles playing “themselves”; some are done with some team roles, played by actual clinicians (but who are scripted confederates). In addition, sometimes these roles are performed by clinician-actors who are not playing their real role, and sometimes by nonclinical actors who are trained and scripted. Here, our results address the one method that we used; therefore, we cannot necessarily generalize our results to the various different ways that simulations are done. There is potential utility in using badges that identified the role, especially when participants are playing many different roles. However, the simulations then conflate the role to be played, with the ability or inability of the individuals to credibly act in those roles, and thence also with the presence or absence of an identifying badge.

The situational awareness during simulation depends not only on environmental factors but also on uncontrollable personal factors. Although the maintained commitment of participants during simulation depends on their situational awareness during the scenario, establishing a safe container is probably more important. Establishing a psychologically safe context by clarifying expectations, and establishing a “fiction contract” with participants, allows learners to engage actively in simulation despite possible disruptions to that engagement such as unrealistic aspects of the simulation.^8^^,^^9^

### Lessons learned

Situational awareness is crucial to optimal performance, both in clinical situations and simulated environments. While multiple factors influence participants' perception of the environment, it is difficult to grasp which ones predominate. Through our study, we attempted to identify if wearing identification tags significantly influences participants' situational awareness in simulated high stakes clinical scenarios. Our results, keeping in mind the limitations of the study, seem to indicate that identification badges do not play a significant role in participants' perception of their environment, but rather their value is influenced and should be interpreted in the context of the environment, the clinical objectives, the different actors involved and the evolution in time.

### Conflicts of interest

The authors declare that they have no conflict of interest.
